# Application of Pharmacokinetic Prediction Platforms in the Design of Optimized Anti-Cancer Drugs

**DOI:** 10.3390/molecules27123678

**Published:** 2022-06-08

**Authors:** Tyler C. Beck, Kendra Springs, Jordan E. Morningstar, Catherine Mills, Andrew Stoddard, Lilong Guo, Kelsey Moore, Cortney Gensemer, Rachel Biggs, Taylor Petrucci, Jennie Kwon, Kristina Stayer, Natalie Koren, Jaclyn Dunne, Diana Fulmer, Ayesha Vohra, Le Mai, Sarah Dooley, Julianna Weninger, Yuri Peterson, Patrick Woster, Thomas A. Dix, Russell A. Norris

**Affiliations:** 1 College of Medicine, Medical University of South Carolina, Charleston, SC 29425, USA; beckt@musc.edu (T.C.B.); morningj@musc.edu (J.E.M.); stoddaan@musc.edu (A.S.); 2 Department of Drug Discovery and Biomedical Sciences, Medical University of South Carolina, Charleston, SC 29425, USA; millscat@musc.edu (C.M.); petersy@musc.edu (Y.P.); woster@musc.edu (P.W.); dixta@musc.edu (T.A.D.); 3 Department of Regenerative Medicine and Cell Biology, Medical University of South Carolina, Charleston, SC 29425, USA; springskc@g.cofc.edu (K.S.); guol@musc.edu (L.G.); moorkels@musc.edu (K.M.); gensemer@musc.edu (C.G.); biggsr@musc.edu (R.B.); pettruct@musc.edu (T.P.); kwonhye@musc.edu (J.K.); stayer@musc.edu (K.S.); korenn@musc.edu (N.K.); dunneja@musc.edu (J.D.); fulmerd@musc.edu (D.F.); vohra@musc.edu (A.V.); mail@musc.edu (L.M.); dooleys@musc.edu (S.D.); bowmanjc@musc.edu (J.W.)

**Keywords:** MEK1, machine learning, toxicity, cancer, drug discovery, drug development

## Abstract

Cancer is the second most common cause of death in the United States, accounting for 602,350 deaths in 2020. Cancer-related death rates have declined by 27% over the past two decades, partially due to the identification of novel anti-cancer drugs. Despite improvements in cancer treatment, newly approved oncology drugs are associated with increased toxicity risk. These toxicities may be mitigated by pharmacokinetic optimization and reductions in off-target interactions. As such, there is a need for early-stage implementation of pharmacokinetic (PK) prediction tools. Several PK prediction platforms exist, including pkCSM, SuperCypsPred, Pred-hERG, Similarity Ensemble Approach (SEA), and SwissADME. These tools can be used in screening hits, allowing for the selection of compounds were reduced toxicity and/or risk of attrition. In this short commentary, we used PK prediction tools in the optimization of mitogen activated extracellular signal-related kinase kinase 1 (MEK1) inhibitors. In doing so, we identified MEK1 inhibitors with retained activity and optimized predictive PK properties, devoid of hERG inhibition. These data support the use of publicly available PK prediction platforms in early-stage drug discovery to design safer drugs.

## 1. Introduction

Cancer is the second most common cause of death in the United States, accounting for 602,350 deaths in 2020 [1]. Cancer-related death rates have precipitously declined over the past two decades. This decline has been due to successful campaigning for tobacco prevention and smoking cessation; improved screening tools; vaccines, and new anti-cancer drugs. Despite improvements in response rate, progression free survival, and overall survival, newly approved anti-cancer drugs are associated with increased toxicity risk [2,3]. Anti-cancer drugs are intrinsically cytotoxic and commonly riddled with pharmacokinetic (PK) issues, such as low bioavailability; poor solubility; cytochrome P450 (CYP) and hERG inhibition; central nervous system (CNS) permeability; skin sensitization, and hepatotoxicity [4,5]. Additionally, chemotherapeutic drugs typically require high-dose treatments; have low therapeutic indices; possess several off-target interactions and are often used in combination with other anti-cancer drugs, increasing the risk of adverse drug-drug interactions [5]. As such, early assessment of PK parameters is critical in determining the quality of anti-cancer agents and should be employed in the early stages of drug discovery and development.

The drug failure rate is astoundingly high, with less than 10% of drug candidates making it to the market after reaching Phase I clinical trials [6]. Pharmacokinetic issues are the most common reason for drug failure, often due to unexpected toxicity. The use of artificial intelligence (AI) is actively revolutionizing drug discovery, allowing for the discovery of safer, more selective drug candidates with lower associated costs and reduced time to market [7,8,9]. AI provides an affordable means of predicting toxicity at the early stages of drug discovery, enabling the selection of compounds with devoid of PK deficits. In particular, the application of PK prediction tools in the early stage of anti-cancer drug discovery may reduce the burden of unanticipated adverse drug reactions.

In this short commentary, we provide data to support the use of publicly available machine learning based PK prediction tools in the optimization of mitogen activated extracellular signal-related kinase kinase 1 (MEK1) inhibitors. MEK1 inhibitors, in combination with rapid accelerated fibrosarcoma B-type (BRAF) inhibitors, have dramatically improved tumor response rate and progression-free survival in patients with stage III or IV metastatic BRAF-mutated melanoma [10]. Four FDA-approved MEK1 inhibitors exist: trametinib, cobimetinib, selumetinib, and binimetinib. These inhibitors are associated with extensive side-effect profiles, most notably rash, diarrhea, fatigue, peripheral edema, dermatitis, hypertension, and cardiomyopathy [11,12,13]. Additionally, MEK1 inhibitors are often associated with life threatening arrhythmias resulting from off-target hERG inhibition [11,12,13,14,15]. As such, we utilized artificial intelligence screening tools to preferentially select for compounds with favorable PK profiles, placing special emphasis on eliminating hERG inhibition and major cytochrome interactions. We hypothesize novel MEK1 inhibitors devoid of hERG/CYP inhibition can be achieved using a machine learning based workflow.

## 2. Results

**Figure 1:**
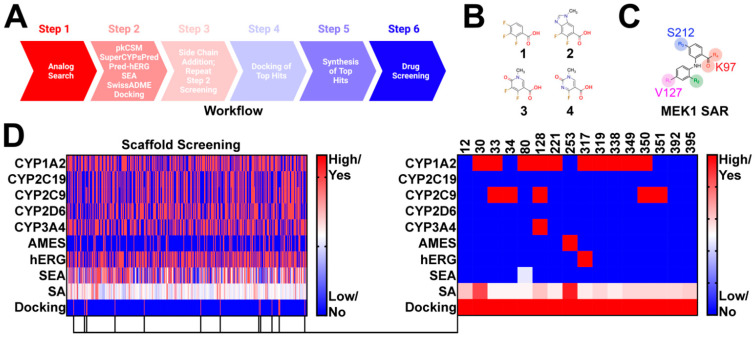
**Identification of Novel synthetic intermediates:** (**A**) workflow used to design novel Norris Lab (NL) compounds. The worklow included 6 steps: (1) analog search using synthetic intermediates derived from FDA-approved MEK1 inhibitors as bait, (2) filtering of synthetic intermediates, followed by PK predictions, (3) novel side-chain addition with repeat PK analysis, (4) docking of hits that emerged from step 3, (5) synthesis of top hits, and (6) drug screening; (**B**) structures used in synthetic intermediates identification. Advanced intermediated were derived from cobimetinib (1), binimetinib and selumetinib (2), TAK-733 (3), and trametinib (4); (**C**) figure highlighting the common structure activity relationship (SAR) shared among nanomolar range MEK1 inhibitors; (**D**) heat map demonstrating PK predictions on the 395 synthetic intermediates discovered. 16 synthetic intermediates were identified; however, only five of the synthetic intermediates were selected based on synthetic accessibility and cost.

Compounds were selected using a six step approach (Figure 1A). Advanced synthetic intermediates were derived from the clinical stage MEK1 inhibitors trametinib, cobimetinib, selumetinib, binimetinib, and TAK-733 (Figure 1B). These intermediates were used as bait in an analog search of the publicly available databases SciFinder-n and ChemSpace (Step 1) (Figure 1A). Several thousand hits were uncovered and subsequently filtered to meet the following criteria: (1) contain a position 1 carboxylic acid, methyl ester, or ethyl ester; (2) possess a leaving group at position 2 for subsequent modification, and (3) conform to the MEK1 structure activity relationship (Figure 1C), with a position 4 or 5 hydrogen bond acceptor. In doing so, 395 hits were selected for further screening in PK prediction platforms and molecular docking (Figure 1D). Several PK prediction platforms were used, including pkCSM, SuperCypsPred, Pred-hERG, Similarity Ensemble Approach (SEA), and SwissADME. Additionally, molecular docking was performed using Shrodinger Glide. pkCSM was used to make predictions on AMES toxicity of advanced synthetic intermediates and gastrointestinal absorption (GIA), CNS permeability (CNSP), volume of distribution (VDSS), and clearance (CL) of hits modified with novel side chains. SuperCypsPred, a highly accurate prediction platform (accuracy: 0.930), was used to make binary predictions on CYP inhibition [7]. Pred-hERG is a highly accurate tool (accuracy: 0.900) used to make predictions on hERG inhibition [16,17]. SEA is a tool that uses chemical similarity to predict off-target protein interactions. As such, SEA was employed to probe synthetic intermediates and structures for potential promiscuity, an undesirable feature that we screened against. Lastly, SwissADME is a tool capable of making predictions on PK parameters, as well as the synthetic accessibility of structures. We used SwissADME to preferentially select for compounds that are synthetically feasible. Of the 395 synthetic intermediates screened, 16 were selected for further study. These synthetic intermediates possess low CYP and hERG inhibition liability with few predicted off-target interactions and reasonable synthetic accessibility. Additionally, all selected synthetic intermediates met our docking criteria, which required energetically favorable interactions with serine-212 (S212) and lysine-97 (K97). Five of the 16 hits identified were pursued based on synthetic accessibility and cost.

**Figure 2:**
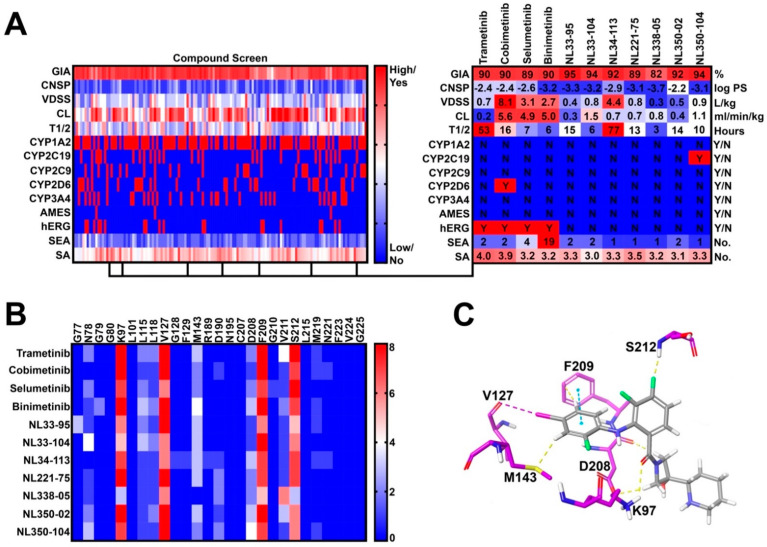
**Virtual compound screen using pharmacokinetic (PK) prediction tools:** (**A**) a total of 27 novel side chains were conjugated to five synthetic intermediates. Compounds were screened for pharmacokinetic properties as previously described. Seven compounds were identified with favorable PK profiles and were selected for molecular docking; (**B**) frequency of drug-allosteric site amino acid interactions quantified from the top 10 most energetically favorable docking poses. Key interactions include lysine-97 (K97), valine-127 (V127), phenylalanine-209 (F209), and serine-212 (S212); and (**C**) visual representation of cobimetinib interacting with key amino acids in the MEK1 allosteric site.

In step 3, 27 novel side chains were conjugated to our five synthetic intermediates, generating 135 compounds for further in silico PK screening (Figure 2A). Compounds were preferentially selected to meet the following criteria: (1) high gastrointestinal absorption (GIA), (2) low CNS permeability (CNSP), (3) low CYP inhibition liability, (4) low hERG inhibition liability, (5) no AMES toxicity, (6) fewer than five off-target interactions, and (7) reasonable synthetic accessibility. As such, seven compounds were selected for molecular docking (Step 4) (Figure 2B). Docking criteria required interactions with lysine-97 (K97), valine-127 (V127), phenylalanine-209 (F209), and serine-212 (S212) (Figure 2C). All Norris Lab (NL) compounds met docking criteria, demonstrating frequent halogen bond interactions with V127, pi-stacking interactions with F209, and hydrogen bonding with S212 and K97 (Figure 2B,C). As such, all seven NL compounds were synthesized for further in vitro testing.

**Figure 3:**
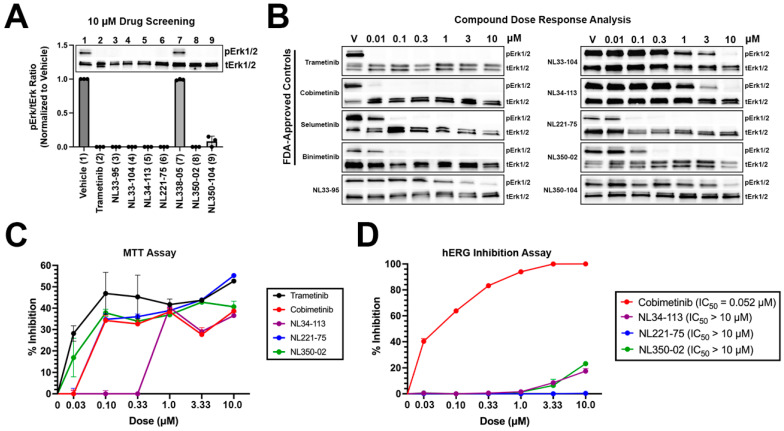
**In vitro screening of novel NL compounds:** (**A**) hit compounds were screened at 10 micromolar concentrations for 24 h. All but one compound demonstrated significant activity and were selected for a dose response analysis; (**B**) compounds were screened at increasing concentrations to assay for activity. NL221-75 and NL350-02 were as potent as FDA-approved controls, demonstrating low nanomolar range activity; (**C**) MTT A375 cells were treated with increasing concentrations of test items and cell proliferation was determined at 24 h using the MTT method. All compounds demonstrated dose-dependent activity in preventing cell proliferation. Experimental compounds NL221-75 and NL350-02 were as effective as FDA-approved controls in preventing cell proliferation. Data are plotted as percent inhibition of proliferation; and (**D**) hERG inhibition experiments performed on CHO-cells. Cobimetinib demonstrated low nanomolar inhibition of hERG. NL34-113, NL221-75, and NL350-02 did not inhibit hERG at the concentrations tested.

NL compounds and controls were screened in A375 malignant melanoma cells at 10 micromolar (μM) concentrations for 24 h (Figure 3A). Trametinib, as well as six NL compounds (NL33-95, NL33-104, NL34-113, NL221-75, NL350-02, and NL350-104), demonstrated significant activity at 10 μM doses. One NL compound, NL338-05, did not demonstrate activity at 10 μM. The six NL compounds with activity, as well as the four FDA-approved MEK1 inhibitors, were selected for a dose response analysis, assessing activity at 0.01, 0.1, 0.3, 1, 3, and 10 μM concentrations (Figure 3B). Trametinib, a MEK1 inhibitor with a cell-free IC_50_ in the picomolar range demonstrated the most potent activity of compounds tested, completely preventing MEK1-induced activation of extracellular signal-regulated protein kinase (ERK1/2) at 0.01 μM. Similarly, cobimetinib, a low nanomolar range inhibitor of MEK1, demonstrated significant activity at 0.01 μM. Selumetinib and binimetinib demonstrated nanomolar range activity, preventing ERK1/2 phosphorylation at 0.1 μM concentrations. Experimental compounds NL33-95, NL33-104, NL34-113, and NL350-104 demonstrated micromolar range activity, with NL221-75 and NL350-02 demonstrating low nanomolar range activity. NL221-75 and NL350-02 were as potent as FDA-approved controls in preventing ERK1/2 activation. Next, we performed an MTT (methylthiazolyldiphenyl-tetrazolium bromide) assay to quantify cell proliferation. A375 cells were treated with increasing concentrations of compounds and cell proliferation was determined at 24 h. All compounds tested exhibited a dose-dependent response in preventing proliferation. NL221-75 and NL350-02 were as effective in preventing cell proliferation as the FDA-approved controls trametinib, cobimetinib, and selumetinib (Figure 3C). Lastly, a hERG inhibition assay was performed to validate our in silico predictions. As predicted, cobimetinib inhibited hERG at low nanomolar concentrations (IC_50_ = 52 nM) (Figure 3D). None of the NL-compounds tested inhibited hERG. These data confirm our computational predictions, establishing a reduced cardiotoxicity potential associated with our novel NL-compounds.

## 3. Discussion

On average, it costs $2.6 billion USD and takes 10 to 15 years to develop an anti-cancer drug [6,18,19]. These costs are a result of high drug attrition rates in clinical stage development [6]. The most common reason for clinical stage failure is poor drug pharmacokinetics. As such, there is a need for early-stage implementation of pharmacokinetic prediction tools. Artificial intelligence and machine learning-based tools can examine compound libraries and predict molecular interactions. These tools can be used to identify patterns and manufacture predictions in a cost-effective and time-efficient manner. PK prediction platforms reduce the risk of unexpected adverse events.

Anti-cancer drugs commonly target pathways that are vital for cell survival and homeostasis. These drugs typically possess a narrow therapeutic index, emphasizing the need to consider pharmacokinetics, off-target interactions, and potential drug-drug interactions. In this short communication, we highlight a successful example of implementing PK prediction tools at an early stage to aid in lead compound selection. pkCSM was used to assess gastrointestinal absorption, half-life, and CNS permeability. Issues with gastrointestinal absorption, oral bioavailability, and a lack of CNS permeability are commonly observed in anti-cancer drug discovery and development. Platforms such as pkCSM and SwissADME, among other computational tools, may be employed to screen hit compounds for potential PK deficits. We selected for compounds without CNS permeability. The decision to do so was based on our target indication; side-effect profile associated with CNS inhibition of MEK1, and the plan to explore topical or injectable use for peripheral indications. We used SuperCypsPred to make binary predictions on CYP inhibition. Major cytochromes, such as CYPs 3A4, 2D6, 2C9, 2C19, and 1A2, are responsible for the metabolism of 80% of FDA-approved drugs, including chemotherapeutics [9]. Screening for potential interactions may reduce the risk of adverse drug reactions. Pred-hERG 4.2 was used to make predictions on hERG inhibition, which is known to precipitate cardiac arrhythmias and significantly contribute to late pre-clinical and clinical stage failure of drugs [20,21]. MEK1 inhibitors commonly block hERG and as such, we strictly selected for compounds devoid of hERG inhibition. The chemical similarity ensemble approach, SEA, was used to make predictions on potential off-target interactions. Off-target interactions, particularly with kinase inhibitors, can lead to significant toxicities and should be screened for during pre-clinical drug development. SEA relates proteins by correlating chemical similarity among their ligands [22,23]. SEA can screen large compound databases and build cross-target similarity maps in real time to predict potential drug-protein interactions. Our compounds were screened using SEA to preferentially select for synthetic intermediates that have reduced potential for problematic off-target interactions. Lead compounds NL221-75 and NL350-02 are predicted to have fewer than two predicted off target-interactions, demonstrating significant selectivity for MEK1. Lastly, SwissADME was used to examine the synthetic accessibility and drug-likeness of our compounds. Most of our compounds were synthetically accessible and did not violate Lipinski’s Rule of Five.

In vitro data from this study confirm computational predictions on hERG inhibition and activity. Six of the seven NL-compounds tested demonstrated activity in preventing ERK1/2 phosphorylation in A375 cells. NL221-75 and NL350-02 were as potent as FDA-approved controls in preventing the activation of ERK1/2. None of the three NL-compounds selected for hERG inhibition studies demonstrated activity. In contrast, Cobimetinib, an FDA-approved MEK1 inhibitor with shared chemical features with our derivatives, demonstrated low nanomolar range activity against hERG (IC_50_ = 52 nM). These data confirm the discovery of novel MEK1 inhibitors with retained activity in vitro and reduced liability of cardiotoxicity. These data validate publicly available PK prediction platforms and further support their use in early-stage drug discovery and development.

### 3.1. Conclusions

We provide data supporting the use of publicly available PK prediction platforms. The development of novel anti-cancer drugs has revolutionized the field of oncology, improving patient outcomes. PK optimization of anti-cancer drugs will reduce the risk of unanticipated adverse events and improve patient outcomes. Our data provide a novel workflow to be used in the identification of compounds with desirable pharmacokinetic properties. This workflow includes cutting-edge machine learning-based platforms capable of making accurate and robust predictions on PK parameters. These data validate the predictive value of these tools and support their use in early-stage drug discovery and development.

### 3.2. Limitations

Moving forward, we plan to screen both NL221-75 and NL350-02 for in vivo efficacy in a mouse model of melanoma and for in vitro interactions with major cytochromes and additional kinases. One limitation of this study is that, currently, we are unable to disclose structural or synthetic information relating to our novel NL-compounds due to intellectual property (IP) considerations. Chemical structures and syntheses data for all NL-derivatives will be deposited to PubChem in the coming months following IP-filing. The purpose of this article is to emphasize the need for early-stage assessment of pharmacokinetic properties using in silico and in vitro approaches. Further pre-clinical development is required to confirm in vivo activity and PK of NL-derivatives relative to FDA-approved controls.

## 4. Materials and Methods

### 4.1. Computational Analyses

Advanced synthetic intermediates of the nanomolar range MEK1 inhibitors trametinib, cobimetinib, selumetinib, binimetinib, and TAK-733 were converted to SMILES codes using Chem Info (http://www.cheminfo.org/flavor/malaria/Utilities/SMILES_generator___checker/index.html accessed on 22 February 2022). SMILES codes were input as bait in ChemSpace (https://chem-space.com/search accessed on 22 February 2022) and SciFinder-n (https://scifinder-n.cas.org/ accessed on 22 February 2022). Hit synthetic intermediates were converted to SMILES codes and submitted for analysis in pkCSM (http://biosig.unimelb.edu.au/pkcsm/prediction accessed on 22 February 2022), SuperCYPsPred (http://insilico-cyp.charite.de/SuperCYPsPred/ accessed on 22 February 2022), Pred-hERG 4.2 (http://predherg.labmol.com.br/ accessed on 22 February 2022), SEA (https://sea.bkslab.org/ accessed on 22 February 2022), and SwissADME (http://www.swissadme.ch/index.php accessed on 22 February 2022). Additionally, structures were converted to Mol.2 files for docking in Schrodinger Glide (New York, NY, USA). Using Schrodinger Glide, coordinates for MEK1 enzyme (PDB: 4l mn) were prepared by removal of crystallographic waters and ions, correction of structural incompletions, addition of partial charges, and protonation at physiologic pH. The active site was defined using the Grid Generation tool. The co-crystalized ligand cobimetinib was used to determine the receptor grid. Compounds were prepared using LigPrep, a tool that takes 2D or 3D structures and produce the corresponding low-energy 3D structures for docking in Glide. In some cases, LigPrep produced multiple output structures for each input structure by generating different protonation states, stereochemistry, tautomers, and ring conformations. These structures were saved as Maestro files and submitted for docking. The top 10 most energetically favorable docking poses were used for analysis. Selected synthetic intermediates were further modified using 27 proprietary side chains. The computational tools described above were repeated in the analysis of the 135 compounds generated.

### 4.2. Cell Culture and Drug Treatment

Human A375 metastatic melanoma cells (ATCC (Manassas, VA, USA); No. CRL-1619IG-2) were cultured in Gibco Dulbecco’s Modified Eagle Medium (DMEM) (ATCC; No. 30-2002) using 10% fetal bovine serum and 1% Penicillin-Streptomycin. Cells were plated at a confluency of 5 × 10^5^ per 35 mm well 24 h before treatment. For initial drug screening, cells were treated with vehicle, trametinib (ChemShuttle (Burlingame, CA, USA); No. 100836, 40 nM), or NL-compounds at 10 μM for 24 h. Compounds were dissolved in DMSO and vehicle treated cells received DMSO in media. For dose response analyses, cells were treated with vehicle or drug (trametinib or NL-compounds) at varying concentrations (0.01, 0.1, 0.3, 1, 3, and 10 μM) for 24 h. Following 24-h treatment, cells were then lysed using 1× RIPA lysis buffer containing 1% protease/phosphatase inhibitor, sonicated, and stored at −80 °C until further use.

### 4.3. Western Blot Analysis

Tissue and cells were homogenized in 1× RIPA with 1% protease/phosphatase inhibitor, followed by sonication. Samples were prepared with 2× SDS Page buffer (125 mM Tris pH7, 4% SDS, 0.2% bromophenol blue, 20% glycerol, 5% β-mercaptoethanol) and boiled at 98 °C for 5 min prior to loading. Proteins were separated in 4–20% Mini-PROTEAN TGX Stain-Free Protein Gels (Bio-rad (Hercules, CA, USA), #456-8093) and transferred to Trans-Blot Turbo Mini Nitrocellulose Transfer Packs (Bio-rad, #170-4158). Membranes were blocked in 5% nonfat milk (Biorad, #170-6404) diluted in 1× Tris Buffered Saline, 0.1% Tween 20 (TBST) for 1 h and incubated in primary antibodies at 4 °C overnight with rotation. Membranes were rinsed 5 times with TBST and incubated at room temperature for 1 h in secondary antibody. The membranes were then rinsed 5 times in TBST for 10 min each and imaged on a Bio-Rad ChemiDoc MP system with SuperSignal West Femto Maximum Sensitivity Substrate (ThermoFisher (Waltham, MA, USA), #34096). Primary antibodies were used at 1:1000 and included p-p44/p42 MAPK (t202/y204) (phospho-ERK1/2) (Cell Signaling (Danvers, MA, USA); No. 4270S) and p44/42 MAPK (ERK1/2) (Cell Signaling; No. 4695S. Secondary anti-rabbit IgG HRP antibody (Sigma (Burlington, MA, USA); No: A9169) was used at 1:7500. Ponceau S (Millipore Sigma; No: P7170) was performed as need for protein normalization.

### 4.4. MTT Cell Proliferation Assay

An MTT assay (Abcam (Cambridge, UK); No: ab211091) was performed following the manufacturer’s instructions. A375 cells were plated in a 96-well plate at a density of 5000 cells/well for 24 h before treatment. Cells were then treated with vehicle or drug (trametinib, cobimetinib, or NL-compounds) at varying concentrations (0.01, 0.1, 0.3, 1, 3, and 10 μM) for 24 h. Following 24-h treatment, drug containing media was exchanged with serum-free DMEM (50 μL/well) and MTT reagent (50 μL/well) and incubated at 37 °C for 3 h. After incubation, MTT solvent (150 μL/well) was added. Plates were wrapped with foil and placed on an orbital shaker for 15 min. Absorbance was read at 590 nm using a BioTek plate reader (BioTek (Hong Kong, China); ELx800). Triplicate readings were measured and averaged for each sample. Readings from culture media-only treated wells were subtracted from test item readings. Cell proliferation was measured using the following formula:

(1) $$Cell\;Proliferation=\frac{\left(Control-Sample\right)}{Control}$$

### 4.5. hERG Inhibition Assay

hERG potassium channel assays were performed by Eurofins St. Charles examining six concentrations (0, 0.1, 0.3, 1, 3, and 10 μM) in CHO-cells, per their publicly available method [24]. The parameters measured were the maximum tail current evoked on stepping to 40 mV and ramping back to −80 mV from the test pulse. All data were filtered for seal quality, seal drop, and current amplitude. The peak current amplitude was calculated before and after compound addition and the amount of block was assessed by dividing the Test compound current amplitude by the Control current amplitude. Control data is the mean hERG current amplitude collected 15 s at the end of the control period; Test compound data is the mean hERG current amplitude collected 15 s at the end of test concentration application for each concentration. All compounds were tested in the presence of 0.1% Pluronic F-68 Non-Ionic Surfactant. After whole cell configuration is achieved, the cell is held at −80 mV. The cell is depolarized to +40 mV for 500 ms and then to −80 mV over a 100 ms ramp to elicit the hERG tail current. This paradigm is delivered once every 8 s to monitor the current amplitude.

## Data Availability

Data are available online [25].

## Sample Availability

Samples of the compounds NL221-75 and NL350-02 are available from the authors.
